# Changes in body weight, body composition and cardiovascular risk factors after long-term nutritional intervention in patients with severe mental illness: an observational study

**DOI:** 10.1186/1471-244X-11-31

**Published:** 2011-02-18

**Authors:** Maria Hassapidou, Konstantina Papadimitriou, Niki Athanasiadou, Valasia Tokmakidou, Ioannis Pagkalos, George Vlahavas, Fotini Tsofliou

**Affiliations:** 1 Department of Nutrition and Dietetics, School of Food Technology and Nutrition, Technological Educational Institute of Thessaloniki, Thessaloniki, Greece; 2 Department of Electrical and Computer Engineering, Aristotle University of Thessaloniki, 54 006 Thessaloniki, Greece

## Abstract

**Background:**

Compared with the general population, individuals with severe mental illness (SMI) have increased prevalence rates of obesity and greater risk for cardiovascular disease. This study aimed to investigate the effects of a long term nutritional intervention on body weight, body fat and cardiovascular risk factors in a large number of patients with SMI.

**Methods:**

Nine hundred and eighty-nine patients with a mean ± S.D age of 40 ± 11.7 yrs participated in a 9 mo nutritional intervention which provided personalised dietetic treatment and lifestyle counselling every two weeks. Patients had an average body mass index (BMI) of 34.3 ± 7.1 kg.m^-2 ^and body weight (BW) of 94.9 ± 21.7 kg. Fasted blood samples were collected for the measurement of glucose, total cholesterol, triglycerides and HDL- cholesterol. All measurements were undertaken at baseline and at 3 mo, 6 mo and 9 mo of the nutritional intervention.

**Results:**

Four hundred and twenty-three patients of 989 total patients' cases (42.8%) dropped out within the first 3 months. Two hundred eighty-five completed 6 months of the program and 145 completed the entire 9 month nutritional intervention. There were progressive statistically significant reductions in mean weight, fat mass, waist and BMI throughout the duration of monitoring (p < 0.001). The mean final weight loss was 9.7 kg and BMI decreased to 30.7 kg.m^-2 ^(p < 0.001). The mean final fat mass loss was 8.0 kg and the mean final waist circumference reduction was 10.3 cm (p < 0.001) compared to baseline. Significant and continual reductions were observed in fasting plasma glucose, total cholesterol and triglycerides concentrations throughout the study (p < 0.001).

**Conclusion:**

The nutritional intervention produced significant reductions in body weight, body fat and improved the cardiometabolic profile in patients with SMI. These findings indicate the importance of weight-reducing nutritional intervention in decreasing the cardiovascular risk in patients with SMI.

## Background

Psychiatric patients have a high prevalence of obesity or a greater risk for weight gain due to antipsychotic (neuroleptic) treatment. Recent studies suggest that patients with severe mental illness (SMI) might have an even higher proportion of obesity than individuals in the general population. For example, Dickerson et al. compared 149 psychiatric patients with matched controls and found that prevalence of obesity was twice as high as the general US adult population (men 41 vs. 20% and women 50 vs. 27%) [1]. As early as the mid-1960s, associations between conventional neuroleptic treatment and metabolic abnormalities were reported. Atypical antipsychotics are newer drugs that are increasingly replacing the conventional neuroleptics due to better efficacy and side effects profile. However evidence suggests that some of the atypical antipsychotics may have even greater associations with dramatic weight gain, diabetes and dyslipidemia [2].

It is well demonstrated that excessive body weight is a clearly established factor for type 2 diabetes and cardiovascular disease in the general population. Changes in some glucose and lipid parameters are commonly reported in patients with all forms of severe mental illness (SMI) (psychosis, depression, bipolar disease). These metabolic changes are probably related to a combination of genetic predisposition, lifestyle factors and psychotropic treatments [3]. Moreover, the burden of weight gain may affect compliance with medication which may predispose psychiatric patients in great health risk. Thus, psychiatric patients appear to be at increased risk of high morbidity and mortality [4].

It becomes clearly understood that controlling and decreasing the weight gain of psychiatric patients should be a priority within their treatment program. It is argued that managing obesity in SMI patients is a challenging task as these patients may have impaired attention, motivation and memory that may impair their ability to follow weight loss program. Behavioral approaches that combine reduced dietary intake and increased physical activity are recommend as most favorable and effective strategy for weight management than pharmacological approaches in psychiatric obese population [5]. In healthy overweight and obese individuals life style interventions through diet and exercise produce significant weight loss and reductions in body fat. Recent studies of dietary and behavioral modification interventions have found small significant weight decreases in SMI patients on antipsychotic medication over short-term intervals [6]. Evidence also suggests significant improvements in the metabolic profile of obese psychiatric patients after weight loss interventions [7].

The long-term effects of nutritional interventions on several adiposity parameters and cardiometabolic parameters are not clearly understood. Previous studies have mainly reported the effects of weight loss on body weight and little is known for the effects on body composition. In addition, although metabolic abnormalities are well documented in patients taking antipsychotics [8], the effects of weight loss on metabolic regulation is not clearly described in psychiatric patients. The previous evidence is derived from controlled clinical trials of small number of patients or from a few naturalistic observational studies of inpatients. Thus, more observational studies of large number of psychiatric outpatients are required to assess management of weight gain and of metabolic disorders. In addition, previous conclusions are tempered by the short term duration of the studies and the small sample sizes used in those studies. Therefore the present study aimed to investigate the effects of a long term nutritional intervention on body weight, body composition and cardiovascular risk factors in a large number of patients with severe mental illness.

## Methods

### Subjects with SMI

A total of 989 psychiatric patients were recruited for the study (774 women and 215 men) and gave written informed consent. Patients were recommended to participate in the study by psychiatrists working either privately or in hospital offices in Thessaloniki (Greece). The study was carried out from January 2007 to November 2009. The study has been approved by the ethical committee of the Technological Educational Institute of Thessaloniki (Ref. No 20111). All patients were found competent by an independent psychiatrist, who was not involved in the study, to participate and to follow weight loss intervention at the enrollment visit. All patients continued on treatment with their medication. Antipsychotic drugs were being used by 28% of patients (n = 274), 30% of patients were taking antidepressants (n = 297), 23% of patients were taking both antipsychotics & antidepressants (n = 230) and 19% of patients (n = 288) were taking antipsychotics & antidepressants, as well other types of medication (e.g. acholytic, antiparkinson, antiepileptic). Medication was kept constant for every patient.

### Anthropometric measurements

Prior to the baseline assessment, patients visited the dietitian for familiarization with study design and measurements. The dietitian explained the study design and measurements thoroughly and then patients' relevant questions were answered.

At the beginning of the study (baseline-visit A), at 3 mo, 6 mo and 9 mo of the nutritional intervention (visit B, C and visit D respectively), several anthropometric measurements were undertaken to assess the outcome of the nutritional intervention program. All the measurements were carried out by the same two dietitians.

Body weight was measured on a standing scale calibrated to 0.1 kg (Seca digital scale). Body height was measured on a wall-mounted stadiometer. The subjects stood with legs parallel and shoulder-width apart. Waist circumference (WC) was measured at the end of normal expiration at the minimal waist (smallest horizontal circumference above the umbilicus and below the xiphoid process). Hip circumference (HP) was measured around the maximum circumference over the buttocks.

Body Fat was measured by the bioelectrical impedance analysis (BIA, Akern version 1.31). During the 9 mo period, subjects were asked to visit the nutrition unit every 2 wks. At these visits, body weight, waist circumference and body fat were measured by the same dietitian. For patients who dropped out, body weight was recorded and BMI was calculated when the drop out occurred.

### Nutritional intervention

The intervention period lasted 9 months and consisted of 2 phases: a familiarization visit and an intensive 9 month nutritional intervention period. The dietary advice for weight control was given in each patient by a registered dietitian. It was based on a Mediterranean-style diet in combination with personalized healthy nutrition counselling. Each patient received personalized dietary regimen on the basis of dietary history and lifestyle. The dietary regimen was characterized by a moderate consumption of carbohydrates (50-55% of total energy per day) and a high fiber content, 15-20% protein and a fat intake of 30-35% of total energy per day. Moreover, patients were advised to consume fruits, vegetables, whole grains (legumes, rice, maize, and wheat) daily and to increase their consumption of olive oil. The dietary regimen was designed to produce an energy deficit of 500 kcal per week. The patients were visiting the dietitian every two weeks to discuss weight changes and treatment goals.

The Resting Metabolic Rate (RMR) was measured by indirect calorimetry (Fitmate Pro, Cosmed USA Inc.) during their first visit. All patients completed a physical activity record. RMR was multiplied by an activity factor of 1.3-1.5, according to the physical activity level of each patient, and daily energy requirements of each patient were estimated. The intervention program consisted primarily of dietary counseling, physical activity counseling and behavioral interventions in order to aid patients' adherence to a healthy life plan during the nutritional intervention. Counseling sessions were undertaken individually by each patient and included teaching healthful weight management techniques, meal planning, food shopping and preparation, portion control, techniques to differentiate emotional from psychological hunger etc. In terms of physical activity counseling, subjects were instructed to participate in light or moderate exercise at least 30 min 3-5 times per week.

### Biochemical measurements

Biochemical measurements were undertaken at the beginning of the study (baseline-Visit A), at 3 mo (Visit B), 6 mo (Visit C) and 9 mo (Visit D) of the nutritional intervention. Data regarding plasma glucose, total cholesterol, HDL cholesterol and triglycerides were recorded by the dietitian.

### Statistical Analysis

Data are expressed as means and standard deviations (SD). Within-subject paired t-tests compared initial vs end point measures for subjects that completed the 9 mo intervention. Comparisons between completers and drop-outs were performed using independent sample t-tests. In order to compensate for missing data due to withdrawal, the last-observation-carried-forward (LOCF) method was used and paired t-tests were performed against the LOCF data as well. Correlation analysis was also carried out for associations between body weight change and body fat percentage (BF %) change over time (baseline to 3 mo, 6 mo and 9 mo). Statistical significance was taken as *P *< 0.05. The statistical analysis was processed with SPSS 11 for Windows (SPSS, Inc., Chicago, IL, USA).

## Results

### Characteristics of SMI subjects and their baseline condition

**Figure 1:**
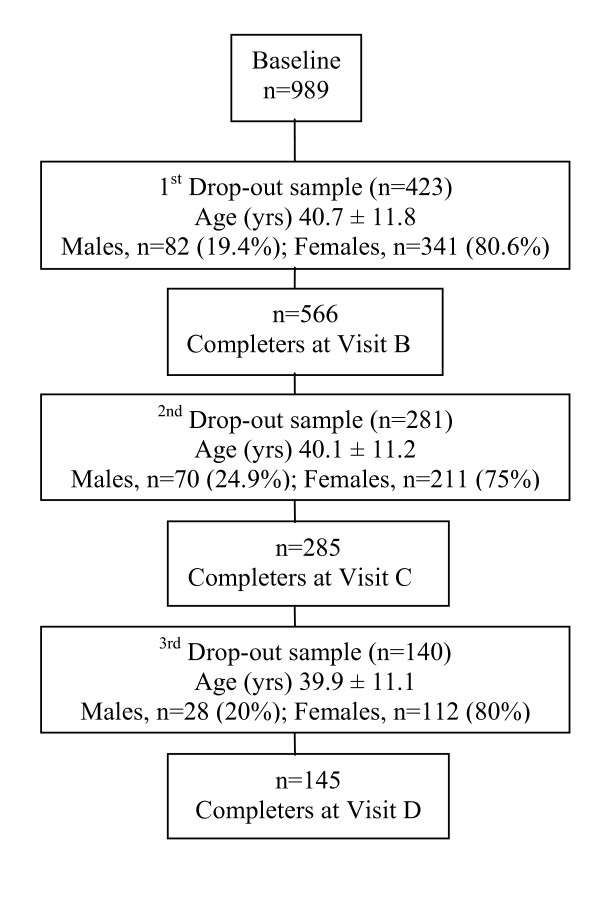
**Participants' Flow**.

Figure 1 presents the participants' flow during the 9 mo nutritional intervention. From the first drop-out sample, 82 subjects were males and 341 subjects were females, with average age 40.7 ± 11.8 y and average body weight 94.9 ± 21.2 kg. From the second drop-out sample, 70 subjects were males and 211 subjects were females, with average age 40.1 ± 11.2 y and average body weight 95.6 ± 23.1 kg (Figure 1). From the 3^rd ^drop-out sample, 28 subjects were males and 112 females. Reasons for dropping out of the study included an inability or unwillingness to continue with the nutritional intervention, family problems, health problems and transportation. Table 1 shows the characteristics of the subjects obtained from the baseline investigation. At baseline, all patients were classified obese (BMI > 30 kg^.^m^-2^) with an average body weight of 94.9 ± 21.7 kg and an average BMI of 34.3 ± 6.9 kg^.^m^-2^. The ratio of men that completed the 9 mo nutritional intervention (completers) was significantly greater than the ratio of women (P = 0.009). No significant differences were found in anthropometric and biochemical characteristics between drop-outs and completers at baseline (P > 0.05) (Table 1).

**Table 1:** Baseline characteristics

		Total subjects		Completers		Drop-outs	p-values
Males (n (%)		215 (21.8%)		44 (30.3%)*		171 (20.3%)	0.009
Females (n (%)		774 (78.4%)		101 (69.7%)		673 (79.7%)	

			n		n		

Age (years)	989	40.2 ± 11.8 (19-80)	145	38.9 ± 12.1	844	40.4 ± 11.5	0.14
Weight (kg)	989	94.9 ± 21.7	145	95.5 ± 21.6	844	94.8 ± 21.7	0.70
Height (m)		1.66 ± 0.09					
BMI (kg^.^m^-2^)		34.3 ± 6.9	145	34.4 ± 7.1	844	34.4 ± 6.9	0.97
Waist (cm)	974	108.9 ± 17.5	144	109.8 ± 18.3	832	108.8 ± 17.4	0.52
Hip (cm)	974	118.4 ± 34.1	144	117.3 ± 11.9	832	117.4 ± 11.5	0.92
Waist/Hip ratio		0.92 ± 0.12		0.94 ± 0.12		0.93 ± 0.11	0.13
Fat mass (%)	803	38.3 ± 8.03	121	37.4 ± 8.13	682	38.4 ± 8.02	0.20
Fat mass (kg)		36.8 ± 13.7		36.1 ± 14.4		36.9 ± 13.5	0.54
RMR	776	1608 ± 439.9	125	1644 ± 408	651	1601 ± 446	0.32
Total Cholesterol (mg/dl)	867	209.4 ± 41.4	139	212.1 ± 43.9	728	208.8 ± 40.9	0.40
HDL-Cholesterol (mg/dl)	755	49.9 ± 14.9	120	50.2 ± 18.6	635	49.9 ± 14.2	0.89
Triglycerides (mg/dl) S	857	151.6 ± 107.8	139	161 ± 114	718	150 ± 107	0.28
Glucose (mg/dl)	884	97.8 ± 21.8	141	98.7 ± 26.5	743	97.6 ± 20.8	0.58

Values are mean ± SD							

### Effect of the nutritional intervention on body composition

Table 2 shows the change in adiposity parameters from baseline to 9 mo of the nutritional intervention in completers and drop-outs. Body weight, BMI, waist and hip decreased significantly from baseline to 3 mo, 6 mo and 9 mo of the intervention in both completers and drop-outs (P < 0.001). In addition, body fat %, body fat mass (kg) decreased significantly at 3 mo, 6 mo and 9 mo of the nutritional intervention relative to baseline in completers (P < 0.001). Baseline measurements of weight and BMI were not significantly different between completers and drop-outs (Table 2). Completers at visit B (3 mo) and visit C (6 mo) had significantly lower weight and BMI than patients who dropped out before visit B and visit C, respectively. Weight and BMI were not significantly different between completers at visit D (9 mo) and patients who dropped out before visit D. The average change of weight and BMI, however, was significantly higher in completers than drop-outs at 9 mo (Δ (weight) 9.7 ± 8.4 vs 5.9 ± 6.2 respectively, P < 0.001; (Δ (BMI) 3.6 ± 3.0 vs 2.1 ± 2.2 respectively, P < 0.001 ). RMR decreased significantly in completers at visit B and C compared to baseline (P < 0.001) (Table 2). The effect of nutritional intervention on body weight and body composition was confirmed when LOCF analysis was performed (Table 3). There were positive associations between change in body weight and BF % change in SMI patients (Visit A to Visit B, r = 0.46 (P < 0.001); Visit A to Visit C, r = 0.46 (P < 0.001); Visit A to Visit C, r = 0.62 (P < 0.001). There was no significant difference in weight loss between patients receiving different psychotropic medication (P > 0.05).

**Table 2:** Changes in parameters of adiposity during the 9 mo nutritional intervention

	Visit A (Baseline) vs Visit B (3 mo)		Visit A (Baseline) vs Visit C (6 mo)		Visit A (Baseline) vs Visit D (9 mo)	
	Completers	Drop-outs (before visit B)	Completers	Drop-outs (before visit C)	Completers	Drop-outs (before visit D)

Weight(Kg)	94.9 ± 22.1 (n = 566)	94.9 ± 21.2 (n = 423)	94.3 ± 20.9 (n = 285)	95.6 ± 23.1 (n = 281)	95.1 ± 21.9 (n = 145)	93.4 ± 19.8 (n = 140)
	90.6 ± 21.2^*, a^	94.5 ± 21.4*	86.8 ± 19.3^†, b^	92.4 ± 22.4^†^	85.5 ± 19.4^††^	87.4 ± 18.8^††^
BMI (kg^.^m^-2^)	34.3 ± 7.1 (n = 554)	34.5 ± 6.8	34.1 ± 6.9 (n = 282)	34.4 ± 7.3	34.3 ± 7.2 (n = 144)	33.9 ± 6.5
	32.8 ± 6.8^*, a^	34.4 ± 6.9*	31.5 ± 6.4^†, b^	33.3 ± 7.2^†^	30.6 ± 6.2^††^	31.8 ± 6.3^††^
Waist (cm)	108.3 ± 17.6 (n = 540)		108.5 ± 17.6 (n = 270)		109.4 ± 18.7 (n = 144)	
	103.7 ± 17.1*		100.8 ± 16.3^†^		99.1 ± 17.9^††^	
Hip (cm)	119.2 ± 44.6 (n = 540)		116.8 ± 11.3 (n = 270)		116.9 ± 12.3 (n = 140)	
	113.5 ± 11.3*		110.2 ± 10.2^†^		108.4 ± 10.6^††^	
Body Fat (%)	38.4 ± 8.1 (n = 275)		37.7 ± 8.4 (n = 121)		36.9 ± 8.6 (n = 50)	
	36.2 ± 8.3*		31.7 ± 11.7^†^		30.5 ± 10.3^††^	
Body Fat(kg)	36.4 ± 14.1 (n = 275)		35.9 ± 13.9 n = 121)		35.8 ± 14.2 (n = 50)	
	32.7 ± 13.3*		30.1 ± 12.0^†^		27.7 ± 11.3^††^	
RMR	1563.0 ± 391.6 (n = 107)		1567.2 ± 383.5 (n = 63)		1642.1 ± 520.0 (n = 21)	
	1469.9 ± 443.3*		1432.9 ± 452.5^†^		1430.5 ± 457.0	

Values are means ± SD. *n *refers to number of adiposity measurements obtained in each visit B, C and D, consequently the same number of baseline measurements is used for the comparisons. Significance differences were determined by paired *t-*tests; *P < 0.001 for the difference between baseline and visit B; ^†^*P *< 0.001 for the difference between baseline and visit C, ^††^*P *< 0.001 for the difference between baseline and visit D. Symbols ^a, b ^show significant differences by independent t-tests between completes and drop-outs at visit B and C respectively (^a ^*P *< 0.01, ^b^*P *= 0.002).

**Table 3:** Last Observation Carried Forward Analysis (LOCF)

	n	Visit A (Baseline)	Visit B (3 mo)	Visit C (6 mo)	Visit D (9 mo)
Weight(Kg)	989	94.9 ± 21.7	92.4 ± 21.3*	91.8 ± 21.3^†^	91.7 ± 21.3 ^††^
BMI (kg^.^m^-2^)	989	34.3 ± 6.9	33.5 ± 6.9*	33.3 ± 6.9^†^	33.2 ± 6.9^††^
Waist (cm)	974	108.9 ± 17.5	106.4 ± 17.3*	105.8 ± 17.6^†^	105.5 ± 17.8^††^
Hip (cm)		118.4 ± 34.1	115.23 ± 11.4*	114.6 ± 11.5^†^	114.5 ± 11.6^††^
Body Fat (%)	803	38.2 ± 8.0	37.5 ± 8.2*	37.2 ± 8.3	37.1 ± 8.3^††^
Body Fat (kg)		36.8 ± 13.7	35.5 ± 13.6*	35.1 ± 13.6	34.9 ± 13.6^††^
RMR (kcal)	776	1608.0 ± 439.9	1600.6 ± 437.6	1596.9 ± 439.4^†^	1595.9 ± 436.3^††^
Total Cholesterol (mg/dl)	867	209.4 ± 41.4	208.1 ± 40.9*	207.5 ± 40.9^†^	207.2 ± 40.9^††^
HDL-Cholesterol (mg/dl)	755	49.9 ± 14.9	49.8 ± 13.9	49.8 ± 13.8	49.7 ± 13.8
Triglycerides (mg/dl)	857	151.6 ± 107.8	150.2 ± 104.1	149.4 ± 103.3^†^	148.0 ± 101.7^††^
Glucose (mg/dl)	884	97.8 ± 21.8	97.3 ± 20.5*	97.3 ± 20.6^†^	97.2 ± 20.4^††^

Significance differences were determined by paired *t-*tests; *P < 0.001 for the difference between baseline and visit B; ^†^*P *< 0.001 for the difference between baseline and visit C, ^††^*P *< 0.001 for the difference between baseline and visit D

### Effects of the nutritional intervention on biochemical parameters

Table 4 shows the change in plasma glucose and plasma lipid concentrations. Fasting plasma glucose concentrations and total cholesterol concentrations decreased significantly from baseline to 3 mo, 6 mo and 9 mo of the intervention (P < 0.05, P < 0.001, P < 0.001, respectively). Fasting plasma triglycerides concentrations decreased significantly at 6 mo and 9 mo of the nutritional intervention compared to baseline (P < 0.001). The nutritional intervention produced a small decrease in HDL-cholesterol compared to baseline but this was not statistically significant (P > 0.05) (Table 4). The effect of nutritional intervention on plasma glucose and plasma lipids was confirmed when LOCF analysis was performed (Table 3).

**Table 4:** Change in biochemical parameters during the 9 mo nutritional intervention

		Visit A (Baseline) vs Visit B (3 mo)		Visit A (Baseline) vs Visit C (6 mo)		Visit A (Baseline) vs Visit D (9 mo)
	n		n		n	
Total Cholesterol (mg/dl)	136	214.8 ± 42.1	66	214.3 ± 44.2	25	215.3 ± 51.1
		206.8 ± 39.9*		200.6 ± 43.3^†^		190.4 ± 44.2^††^
HDL-Cholesterol (mg/dl)	54	47.9 ± 24.6	38	48.7 ± 28.3	17	50.3 ± 12.5
		45.1 ± 12.5		43.9 ± 10.3		47.5 ± 10.2^††^
Triglycerides (mg/dl)	135	162.4 ± 113.7	65	175.8 ± 112.9	25	213.1 ± 167.1
		153. 3 ± 89.9*		158.4 ± 91.8^†^		135.3 ± 74.4^††^
Glucose (mg/dl)	139	98.3 ± 26.6	68	99.1 ± 20.2	25	100.8 ± 17.6
		95.3 ± 18.5*		95.3 ± 15.9^†^		96.5 ± 16.2^††^

Values are means ± SD. *n *refers to number of biochemical measures obtained in each visit B, C and D. Significance differences were determined by paired *t-*tests; *P < 0.001 for the difference between baseline and visit B; ^†^*P *< 0.001 for the difference between baseline and visit C, ^††^*P *< 0.001 for the difference between baseline and visit D.

## Discussion

This study shows that a personalized nutritional intervention is effective in decreasing adiposity and metabolic parameters in patients with severe mental illness. Previous lifestyle interventions have clearly reported weight loss in patients with severe mental illness but these results were derived from small number of patients and over short term intervals [6,9]. The present study used a large sample size and a 9 month nutritional intervention in order to investigate changes on both adiposity and metabolic parameters in patients with severe mental illness.

The present study found a progressive statistically significant decrease in mean adiposity parameters throughout the duration of monitoring compared to baseline. There is a paucity of clinical trials of management of obesity in patients with severe mental illness. The randomized controlled studies found significant weight reductions or modest reductions on body weight in patients taking antipsychotic medication [10-17]. A small number of nonrandomized controlled studies reported significant weight change [18,19], while Ball and colleagues [20] reported no significant weight change between the nonrandomized intervention group and control group. The present study found a mean weight loss at 3 months of 4.3 kg which is in agreement with other studies [11-18]. However, the evidence is poor for the long term effects of nutritional intervention on adiposity parameters. In our study, the mean weight loss of 7.4 kg at 6 months is greater compared to previous open studies [16,21,22]. The mean weight reduction of 9.6 kg at 9 mo was progressive and significant and exceeds the weight loss achieved in previous long term studies with behavioral treatment programs [23,24]. In addition weight loss was also found significant and continual in the drop-outs which probably indicates a general efficacy of the present nutritional intervention. The body weight management in our patients was undertaken with personalized dietetic treatment and lifestyle counseling. Patients were seen by a dietitian who assessed weight changes and treatment goals every two weeks. The greater weight loss in our study might indicate that a personalized nutritional intervention can produce significant weight loss in psychiatric patients who manage to adhere to the nutritional intervention for more than three months.

The present nutritional intervention not only reduced body weight but demonstrated continual significant decrease in body fat mass (kg) and percent of body fat (%) in our patients. Skouroliakou et al. [17] reported significant reduction in fat mass but in the short term. The present decrease in fat mass is demonstrated for the first time in a long term nutritional intervention in SMI patients. The mean fat mass reduction was continual and significant throughout the study (e.g. 6 kg fat mass loss at 3 mo; 5.9 kg fat mass loss at 6 mo and 8 kg fat mass loss at 9 mo). BMI was also significantly decreased verifying the decrease in total body fat and general obesity. Moreover waist circumference, a well documented proxy for visceral obesity [25], was significantly decreased in our patients. Consistent with previous studies [26], weight loss produced a decrease in RMR. These findings in SMI patients are comparable to reduction of obesity-related factors with lifestyle modification within the general obese population [27]. Recent consensus guidelines for patients with severe mental illness recommend the measurements of both BMI and WC to monitor cardiovascular risk factors in this population [28]. The reductions found in waist circumference and body mass in our SMI patients indicate improvements in the risk factors associated with cardiovascular disease.

The reduction in fasting glucose was significant throughout the nutritional intervention compared to baseline. This is important since abnormalities in glucose metabolism have been associated with the use of antipsychotic treatment [29]. The significant reduction in fasting glucose may be primarily due to weight loss since medication was kept constant. Similarly there were significant reductions in total cholesterol and triglycerides during the 9 month nutritional intervention. These reductions in lipids concentrations are also important since psychiatric patients have been shown to have elevated dyslipidemia compared to general population [30]. Both total cholesterol and triglycerides dropped significantly since weight loss became significant throughout the intervention. The present results justify the important use of weight reducing programs and especially of nutritional intervention in the management of metabolic dysregulation in patients with severe mental illness.

### Limitations

By design the present study did not include a control group, so it is unknown whether a similar group of obese patients would have lost or gained weight over the same time period. Ideally, longer term randomized controlled trials are needed to assess the effectiveness of the nutritional interventions. In addition, we can not draw conclusions on the long-term effectiveness of the intervention by means of weight maintenance as a follow-up period was not included. However, the present results are derived from a relatively large sample compared to previous shorter term or longer term studies of small-subject numbers. Another limitation of the present study is the large drop-out. It is recognized that psychiatric disorders can be a significant barrier to weight loss success in obese individuals, thus discontinuance of the study could have been expected. In a meta-analysis of compliance studies, DiMatteo et al. showed that patients with depression had a 3-fold higher rate of noncompliance with medical treatments, including diet recommendations [31]. However, the significant results from LOCF analysis confirm the efficacy of the 9 mo nutritional intervention in terms of successful weight loss and improvement of the metabolic profile in our SMI patients.

## Conclusions

This study has important clinical implication, indicating the effectiveness of a simple nutritional intervention on adiposity and lipid regulation which is important in psychiatric patients who are a high risk group for the development of cardiovascular disease. The present results show that obese patients with severe mental illness can achieve weight control and improve cardiometabolic profile by following a simple personalized nutritional program for 9 months.

## Competing interests

The authors declare that they have no competing interests.

## Authors' contributions

FT contributed to the interpretation of the data, analysis of the results and prepared this manuscript. KP, VT, NA and IP were involved in data collection and analysis of the results. GV was involved in the statistical analysis of the revised manuscript. MH was the principal investigator and assisted in data collection, interpretation of the results and preparation of the manuscript. All authors read and approved the final version of the manuscript.

## Pre-publication history

The pre-publication history for this paper can be accessed here:

http://www.biomedcentral.com/1471-244X/11/31/prepub

## References

[B1] Dickerson FB Brown CH Kreyenbuhl JA Fang L Goldberg RW Wohlheiter K Dixon LB Obesity among individuals with serious mental illness Acta Psychiatr Scand 2006 113 306 313 10.1111/j.1600-0447.2005.00637.x 16638075

[B2] Lean ME Pajonk FG Patients on atypical antipsychotic drugs: another high-risk group for type 2 diabetes Diabetes Care 2003 26 1597 1605 10.2337/diacare.26.5.1597 12716824

[B3] Bushe CJ Bradley AJ Doshi S Karagianis J Changes in weight and metabolic parameters during treatment with antipsychotics and metformin: do the data inform as to potential guideline development? A systematic review of clinical studies Int J Clin Pract 2009 63 1743 1761 10.1111/j.1742-1241.2009.02224.x 19840151

[B4] Bell RC Farmer S Ries R Srebnik D Metabolic risk factors among medicaid outpatients with schizophrenia receiving second-generation antipsychotics Psychiatr Serv 2009 60 1686 1689 10.1176/appi.ps.60.12.1686 19952163

[B5] Werneke U Taylor D Sanders TA Options for pharmacological management of obesity in patients treated with atypical antipsychotics Int J Clin Psychopharmacol 2002 17 145 160 10.1097/00004850-200207000-00001 12131598

[B6] Faulkner G Cohn T Remington G Interventions to reduce weight gain in schizophrenia Cochrane Database Syst Rev 2007 24 CD005148 10.1002/14651858.CD005148.pub2 PMC4164479 17253540

[B7] Ganguli R Behavioral therapy for weight loss in patients with schizophrenia J Clin Psychiatry 2007 68 19 25 10.4088/JCP.0607e14 17539696

[B8] Birkenaes AB Birkeland KI Engh JA Faerden A Jonsdottir H Ringen PA Friis S Opjordsmoen S Andreassen OA Dyslipidemia independent of body mass in antipsychotic-treated patients under real-life conditions J Clin Psychopharmacol 2008 28 132 137 10.1097/JCP.0b013e318166c4f7 18344722

[B9] Alvarez-Jiménez M Hetrick SE González-Blanch C Gleeson JF McGorry PD Non-pharmacological management of antipsychotic-induced weight gain: systematic review and meta-analysis of randomised controlled trials Br J Psychiatry 2008 193 101 107 18669990 10.1192/bjp.bp.107.042853

[B10] Littrell KH Hilligoss NM Kirshner CD Petty RG Johnson CG The effects of an educational intervention on antipsychotic-induced weight gain J Nurs Scholarsh 2003 35 237 241 10.1111/j.1547-5069.2003.00237.x 14562491

[B11] Brar JS Ganguli R Pandina G Turkoz I Berry S Mahmoud R Effects of behavioral therapy on weight loss in overweight and obese patients with schizophrenia or schizoaffective disorder J Clin Psychiatry 2005 66 205 212 10.4088/JCP.v66n0208 15705006

[B12] Evans S Newton R Higgins S Nutritional intervention to prevent weight gain in patients commenced on olanzapine: a randomized controlled trial Aust N Z J Psychiatry 2005 39 479 486 15943650 10.1080/j.1440-1614.2005.01607.x

[B13] Kwon JS Choi JS Bahk WM Yoon Kim C Hyung Kim C Chul Shin Y Park BJ Geun Oh C Weight management program for treatment-emergent weight gain in olanzapine-treated patients with schizophrenia or schizoaffective disorder: A 12-week randomized controlled clinical trial J Clin Psychiatry 2006 67 547 553 10.4088/JCP.v67n0405 16669719

[B14] Weber M Wyne K A cognitive/behavioral group intervention for weight loss in patients treated with atypical antipsychotics Schizophr Res 2006 83 95 101 10.1016/j.schres.2006.01.008 16507343

[B15] Jean-Baptiste M Tek C Liskov E Chakunta UR Nicholls S Hassan AQ Brownell KD Wexler BE A pilot study of a weight management program with food provision in schizophrenia Schizophr Res 2007 96 198 205 10.1016/j.schres.2007.05.022 17628437

[B16] Khazaal Y Fresard E Rabia S Chatton A Rothen S Pomini V Grasset F Borgeat F Zullino D Cognitive behavioural therapy for weight gain associated with antipsychotic drugs Schizophr Res 2007 91 169 77 10.1016/j.schres.2006.12.025 17306507

[B17] Skouroliakou M Giannopoulou I Kostara C Hannon JC Effects of nutritional intervention on body weight and body composition of obese psychiatric patients taking olanzapine Nutrition 2009 25 729 735 10.1016/j.nut.2008.12.009 19286349

[B18] Vreeland B Minsky S Menza M Radler DR Roemheld B A Program for managing weight gain associated with atypical antipsychotics Psychiatr Serv 2003 54 1155 1157 10.1176/appi.ps.54.8.1155 12883145

[B19] Menza M Vreeland B Minsky S Gara M Radler DR Sakowitz M Managing atypical antipsychotic-associated weight gain: 12-month data on a multimodal weight control program J Clin Psychiatry 2004 65 471 477 10.4088/JCP.v65n0404 15119908

[B20] Ball MP Coons VB Buchanan RW A program for treating olanzapine-related weight gain Psychiatr Serv 2001 52 967 969 10.1176/appi.ps.52.7.967 11433117

[B21] Centorrino F Wurtman JJ Duca KA Fellman VH Fogarty KV Berry JM Guay DM Romeling M Kidwell J Cincotta SL Baldessarini RJ Weight loss in overweight patients maintained on atypical antipsychotic agents Int J Obes 2006 30 1011 1016 10.1038/sj.ijo.0803222 16432547

[B22] Lindenmayer JP Khan A Wance D Maccabee N Kaushik S Outcome evaluation of a structured educational wellness program in patients with severe mental illness J Clin Psychiatry 2009 70 1385 1396 10.4088/JCP.08m04740yel 19778494

[B23] Pendlebury J Bushe CJ Wildgust HJ Holt RI Long-term maintenance of weight loss in patients with severe mental illness through a behavioural treatment programme in the UK Acta Psychiatr Scand 2007 115 286 294 10.1111/j.1600-0447.2006.00906.x 17355519

[B24] Poulin MJ Chaput JP Simard V Vincent P Bernier J Gauthier Y Lanctôt G Saindon J Vincent A Gagnon S Tremblay A Management of antipsychotic-induced weight gain: prospective naturalistic study of the effectiveness of a supervised exercise programme Aust N Z J Psychiatry 2007 41 980 989 10.1080/00048670701689428 17999270

[B25] Han TS McNeill G Seidell JC Lean ME Predicting intra-abdominal fatness from anthropometric measures: the influence of stature Int J Obes Relat Metab Disord 1997 21 587 593 10.1038/sj.ijo.0800446 9226490

[B26] Weyer C Pratley RE Sable AD Bogardus C Ravussin E Tataranni PA Energy expenditure, fat oxidation, and body weight regulation: a study of metabolic adaptation to long-term weight change J Clin Endocrinol Metab 2000 85 1087 1094 10.1210/jc.85.3.1087 10720044

[B27] Zhu SK Wang Z Heshka S Heo M Faith MS Heymsfield SB Waist circumference and obesity associated risk factors among whites in the Third National Health and Nutrition Examination Survey: clinical action thresholds Am J Clin Nut 2002 76 743 749 10.1093/ajcn/76.4.743 12324286

[B28] Executive Summary of The Third Report of The National Cholesterol Education Program (NCEP) Expert Panel on Detection Evaluation And Treatment of High Blood Cholesterol In Adults (Adult Treatment Panel III) Expert Panel on Detection, Evaluation, and Treatment of High Blood Cholesterol in Adults JAMA 2001 285 16 2486 2497 11368702 10.1001/jama.285.19.2486

[B29] Scheen AJ De Hert MA Abnormal glucose metabolism in patients treated with antipsychotics Diabetes Metab 2007 33 169 175 10.1016/j.diabet.2007.01.003 17412628

[B30] Monteleone P Martiadis V Maj M Management of schizophrenia with obesity, metabolic, and endocrinological disorders Psychiatr Clin North Am 2009 32 775 794 10.1016/j.psc.2009.08.003 19944883

[B31] DiMatteo M Lepper H Croghan T Depression is a risk factor for noncompliance with medical treatment Arch Intern Med 2000 160 2101 2107 10.1001/archinte.160.14.2101 10904452

